# Curative efficacy of surgical procedures for older patients with femoral neck fracture: a network meta-analysis and systematic review

**DOI:** 10.1186/s13018-022-02914-y

**Published:** 2022-03-02

**Authors:** Lanxin Cui, Shishun Zhao, Heng Tian, Wenlai Guo, Xiaoming Dong

**Affiliations:** 1 College of Mathematics, Jilin University, Changchun, Jilin Province People’s Republic of China; 2 Department of Hand Surgery, The Second Hospital of Jilin University, Changchun, Jilin Province People’s Republic of China; 3 Orthopaedic Medical Center, The Second Hospital of Jilin University, 218 Ziqiang Street, Changchun, 130041 Jilin Province People’s Republic of China

**Keywords:** Femoral neck fracture, Elderly, Meta-analysis

## Abstract

**Background:**

Femoral neck fractures have a higher incidence in older people with poor prognosis, inducing serious social problems. Common treatment methods include total hip arthroplasty, bipolar hemiarthroplasty, double-screw fixation, multiple-screw fixation, and dynamic hip system.

**Methods:**

We searched through four electronic databases, including PubMed, Web of Science, Cochrane Library, and Embase databases, for articles regarding femoral neck fractures, bone screw, and hip prosthesis published up to February 11, 2020. All included articles were used for quality evaluation and data extraction. Extracted data were expressed as odds ratios or weighted mean differences, with 95% confidence intervals. We conducted a network meta-analysis for Harris hip score, complications, 1-year mortality rate, reoperation rate, intraoperative blood loss, and duration of operation using STATA version 16.0 software.

**Results:**

Twenty-two randomized controlled trials and nine cohort studies included in this study involved 3861 patients. Total hip arthroplasty significantly improved the postoperative function of patients with femoral neck fractures. The surface under the cumulative ranking curve value of the Harris hip score for more than 1 year after total hip arthroplasty was 98.2.

**Conclusions:**

This meta-analysis indicated no significant difference in mortality among different treatment groups. Total hip arthroplasty can provide satisfactory outcomes in hip joint function, and double-screw fixation results in the lowest intraoperative risk. In general, total hip arthroplasty results in a lower incidence of adverse events and is especially recommended for patients with femoral neck fractures. This article has been retrospectively registered on the International Platform of Registered Systematic Review and Meta-analysis Protocols (INPLASY) on November 27, 2020. Registration number: INPLASY2020110123.

**Supplementary Information:**

The online version contains supplementary material available at 10.1186/s13018-022-02914-y.

## Introduction

Femoral neck fractures occur below the femoral head and above the base of the femoral neck due to direct or indirect violent force. These fractures generally occur in older adults. Severe complications include femoral head necrosis and fracture nonunion, which seriously affect daily living and health. With an aging population worldwide, this disease has become a serious social problem, and by 2050, it is estimated that 63 million patients will have suffered a hip fracture [1, 2].

Increasing concern has been focused on improving patient postoperative survival rate and quality of life after femoral neck fractures. Indeed, special consideration must be given to improving patient hip function, postoperative complications, and reoperation rate when choosing a treatment method for femoral neck fractures. Such methods commonly include bipolar hemiarthroplasty (BHA), total hip arthroplasty (THA), and internal fixation.

Internal fixation is a widely used method worldwide. Numerous studies have demonstrated that internal fixation results in a higher complication rate and surgical failure rate [3, 4]. Some studies have pointed out that the internal fixation failure rate or reoperation rate in elderly patients is 7–30%. [5]. However, compared with arthroplasty, internal fixation has the advantages of less blood loss, shorter operation time, and lower implant cost. For elderly patients, lower bleeding volume and shorter operation time mean lower intraoperative risk. In recent years, with the development of material science, many scholars have found the potential value of internal fixation. [6]. Some work has confirmed the reliability of internal fixation with special materials for fracture fixation [7, 8].Compared with internal fixation, arthroplasty (BHA or THA) effectively reduces the incidence of postoperative complications and reoperation rates. Today, unipolar arthroplasty is generally deprecated as it causes severe damage to the acetabulum. Therefore, we included only publications regarding bipolar hemiarthroplasty (BHA) in this analysis. Contrasted with THA, BHA has the advantages of a shorter operation time, less blood loss, fewer technical requirements, less economic burden, and lower dislocation rate [4, 6]. Some studies assert that BHA is preferred for older patients with low activity levels or cognitive impairment. However, for patients with high activity levels, BHA has a high risk of complications related to posterior acetabular erosion and reoperation [7, 8]. An artificial hip joint consists of an artificial acetabulum and an artificial femoral head. Patients undergoing THA tend to achieve better hip function, less acetabular erosion, and a lower revision rate [12]. Generally, THA is recommended for older patients with acetabular disease [13]. THA has potential limitations in highly active patients. An increase in the wear of the prosthetic head and pads may lead to aseptic loosening, and revision surgery is required accordingly, leading to an increase in the reoperation rate. Therefore, for patients with a healthy acetabulum, BHA is preferred because of reduced surgical trauma and lower surgical cost.

Numerous meta-analyses and systematic reviews have reported pairwise comparisons between THA and HA, between internal fixation and HA, and between types of internal fixations. However, there is limited evidence from available meta-analyses. Traditional meta-analysis methods cannot thoroughly compare all treatment strategies described in the existing studies. Therefore, the emerging network meta-analysis method is considered for use in this meta-analysis [12, 13]. Jonathan et al. [12] revealed that arthroplasty (THA and HA) has the lowest revision rate among THA, HA, screw fixation, steel plate fixation, and treatments for unthreaded cervical osteosynthesis. In this study, the surgical revision rate was the only outcome indicator. Zhang et al. [13] conducted a Bayesian network meta-analysis to rank five surgical procedures in terms of reoperation, mortality, dislocation, and infection. However, we considered various outcome indicators in this meta-analysis, except for surgical procedures involved in different internal fixations.

We conducted a network meta-analysis of all relevant random evidence and considered several prognostic indicators such as Harris hip score (HHS), complication rate, 1-year mortality rate, reoperation rate, intraoperative blood loss, and operation duration. We also fully discussed and ranked the existing treatment methods for femoral neck fractures, including THA, BHA, double-screw internal fixation, multiple-screw internal fixation, and DHS (dynamic hip screws). Therefore, this study presents comprehensive recommendations for the clinical treatment of femoral neck fractures.

## Materials and methods

### Search strategy

This study was conducted according to the Preferred Reporting Items for Systematic Reviews and Meta-Analyses (PRISMA) statement and AMSTAR (Assessing the Methodological Quality of Systematic Reviews) guidelines.Please see PRISMA checklist in Additional file 1. We searched four databases, including PubMed, Embase, Web of Science, and Cochrane Library, for relevant articles published through February 11, 2020 using the search terms “femoral neck fracture,” “bone screw,” “bone nail,” and “bone plate,” “hip prosthesis,” “THA,” and “hip replacement.” Paper retrieval was performed in PubMed applying the following string: (((((((((((((((((((((((Arthroplasty, Replacement, Hip[Title/Abstract] OR (("arthroplasty, replacement"[MeSH Terms] OR ("arthroplasty"[All Fields] AND "replacement"[All Fields]) OR "replacement arthroplasty"[All Fields] OR ("arthroplasties"[All Fields] AND "replacement"[All Fields]) OR "arthroplasties, replacement"[All Fields]) AND Hip[Title/Abstract])) OR (("arthroplasty"[MeSH Terms] OR "arthroplasty"[All Fields]) AND Hip Replacement[Title/Abstract])) OR Hip Prosthesis Implantation[Title/Abstract]) OR Hip Prosthesis Implantations[Title/Abstract]) OR (("embryo implantation"[MeSH Terms] OR ("embryo"[All Fields] AND "implantation"[All Fields]) OR "embryo implantation"[All Fields] OR "implantation"[All Fields]) AND Hip Prosthesis[Title/Abstract])) OR (Implantations[All Fields] AND Hip Prosthesis[Title/Abstract])) OR (("prosthesis implantation"[MeSH Terms] OR ("prosthesis"[All Fields] AND "implantation"[All Fields]) OR "prosthesis implantation"[All Fields]) AND Hip[Title/Abstract])) OR (("prosthesis implantation"[MeSH Terms] OR ("prosthesis"[All Fields] AND "implantation"[All Fields]) OR "prosthesis implantation"[All Fields] OR ("prosthesis"[All Fields] AND "implantations"[All Fields]) OR "prosthesis implantations"[All Fields]) AND Hip[Title/Abstract])) OR Hip Replacement Arthroplasty[Title/Abstract]) OR (("arthroplasty, replacement"[MeSH Terms] OR ("arthroplasty"[All Fields] AND "replacement"[All Fields]) OR "replacement arthroplasty"[All Fields] OR ("replacement"[All Fields] AND "arthroplasties"[All Fields]) OR "replacement arthroplasties"[All Fields]) AND Hip[Title/Abstract])) OR (("replantation"[MeSH Terms] OR "replantation"[All Fields] OR "replacement"[All Fields]) AND Arthroplasty, Hip[Title/Abstract])) OR (("arthroplasty"[MeSH Terms] OR "arthroplasty"[All Fields] OR "arthroplasties"[All Fields]) AND Hip Replacement[Title/Abstract])) OR Hip Replacement Arthroplasties[Title/Abstract]) OR (("arthroplasty, replacement, hip"[MeSH Terms] OR ("arthroplasty"[All Fields] AND "replacement"[All Fields] AND "hip"[All Fields]) OR "hip replacement arthroplasty"[All Fields] OR ("hip"[All Fields] AND "replacement"[All Fields]) OR "hip replacement"[All Fields]) AND Total[Title/Abstract])) OR (("replantation"[MeSH Terms] OR "replantation"[All Fields] OR "replacement"[All Fields]) AND Total Hip[Title/Abstract])) OR (("arthroplasty, replacement, hip"[MeSH Terms] OR ("arthroplasty"[All Fields] AND "replacement"[All Fields] AND "hip"[All Fields]) OR "hip replacement arthroplasty"[All Fields] OR ("hip"[All Fields] AND "replacements"[All Fields]) OR "hip replacements"[All Fields]) AND Total[Title/Abstract])) OR (("replantation"[MeSH Terms] OR "replantation"[All Fields] OR "replacements"[All Fields]) AND Total Hip[Title/Abstract])) OR Total Hip Replacements[Title/Abstract]) OR Total Hip Replacement[Title/Abstract]) OR (((((((Hip Prosthesis[Title/Abstract] OR Hip Prostheses[Title/Abstract]) OR (("prostheses and implants"[MeSH Terms] OR ("prostheses"[All Fields] AND "implants"[All Fields]) OR "prostheses and implants"[All Fields] OR "prostheses"[All Fields]) AND Hip[Title/Abstract])) OR (("prostheses and implants"[MeSH Terms] OR ("prostheses"[All Fields] AND "implants"[All Fields]) OR "prostheses and implants"[All Fields] OR "prosthesis"[All Fields]) AND Hip[Title/Abstract])) OR Femoral Head Prosthesis[Title/Abstract]) OR Femoral Head Prostheses[Title/Abstract]) OR (("prostheses and implants"[MeSH Terms] OR ("prostheses"[All Fields] AND "implants"[All Fields]) OR "prostheses and implants"[All Fields] OR "prostheses"[All Fields]) AND Femoral Head[Title/Abstract])) OR (("prostheses and implants"[MeSH Terms] OR ("prostheses"[All Fields] AND "implants"[All Fields]) OR "prostheses and implants"[All Fields] OR "prosthesis"[All Fields]) AND Femoral Head[Title/Abstract]))) OR (((Bone Plates[Title/Abstract] OR Bone Plate[Title/Abstract]) OR (("bone plates"[MeSH Terms] OR ("bone"[All Fields] AND "plates"[All Fields]) OR "bone plates"[All Fields] OR "plate"[All Fields]) AND Bone[Title/Abstract])) OR (Plates[All Fields] AND Bone[Title/Abstract]))) OR (((((((Bone Nails[Title/Abstract] OR (("bone nails"[MeSH Terms] OR ("bone"[All Fields] AND "nails"[All Fields]) OR "bone nails"[All Fields] OR "pins"[All Fields]) AND Bone[Title/Abstract])) OR (Pin[All Fields] AND Bone[Title/Abstract])) OR Bone Pin[Title/Abstract]) OR Bone Pins[Title/Abstract]) OR (("nails"[MeSH Terms] OR "nails"[All Fields]) AND Bone[Title/Abstract])) OR (("nails"[MeSH Terms] OR "nails"[All Fields] OR "nail"[All Fields]) AND Bone[Title/Abstract])) OR Bone Nail[Title/Abstract])) OR (((Bone Screws[Title/Abstract] OR Bone Screw[Title/Abstract]) OR (Screw,[All Fields] AND Bone[Title/Abstract])) OR (Screws[All Fields] AND Bone[Title/Abstract]))) AND (((Femoral Neck Fracture[Title/Abstract] OR Femur Neck Fracture[Title/Abstract]) OR Femoral Neck Fractures[Title/Abstract]) OR Femur Neck Fractures[Title/Abstract]). Additionally, all included articles were independently assessed by three researchers by reading the full text. Any disagreement was resolved by the fourth researcher.

### Inclusion and exclusion criteria

The inclusion criteria were as follows: (1) middle-aged and elderly patients with femoral neck fractures of Garden types I to IV; (2) at least one of five surgical methods described (THA, BHA, double-screw internal fixation, multiple-screw internal fixation, DHS); (3) at least one of six outcome indicators described (HHS score, complications, mortality within one year, reoperations, intraoperative blood loss, and duration of surgery); (4) randomized controlled trials or cohort studies; (5) written in the English language.

The exclusion criteria were as follows: (1) basic studies about biomechanics and autopsy; (2) femoral neck fracture (age < 60 years); (3) patients with pathological femoral neck fractures; (4) non-surgical interventions; (4) valid data could not be extracted or converted; (5) case–control, paired analysis, conference abstracts, systematic reviews, and meta-analysis studies.

### Outcome measures

This study included the prognostic indicators of patients with femoral neck fractures as outcome measures.

#### HHS

HHS is a widely used method to evaluate hip joint function. In this study, HHS was used to assess the arthroplasty effect in four aspects: pain, function, deformity, and mobility. A higher score indicated a better hip joint function.

#### Surgical complications

Postoperative complications associated with femoral neck fractures commonly include fixation failure, nonunion, osteonecrosis, infection, and nerve paralysis. The incidence of postoperative complications is a powerful indicator for evaluating the effect of surgery.

#### Reoperation

Because of artificial joint wear, screw dislocation, or serious complications, reoperation may be required in some patients with femoral neck fractures. Reoperation indicates a failure of the initial operation and is an important indicator of the quality of the operation.

#### Mortality

The mortality was calculated by counting the number of deaths within 12 months after surgery.

#### Blood loss

Intraoperative blood loss (mL) was statistically counted. Generally, the intraoperative blood loss is proportional to the risk of death.

#### Operation time

Operation time (min) was statistically counted. Generally, the operation time is proportional to the risk of surgical failure.

Additionally, related factors such as surgical approach, prosthesis model, and demographic data were recorded for further discussion.

### Data extraction and management

Three researchers independently extracted data from all the included studies according to a standard data extraction format. Any disagreement was resolved by discussion with another researcher. In some cases, the standard deviation (SD) was not available. Attempts were made to contact corresponding authors in such cases, but no response was available. Thus, for these cases, we estimated the range or median, or used the method described in the Cochrane Intervention Manual Systematic Evaluation Manual to convert data and estimate the SD from the confidence interval (CI) [16].

### Statistical analysis

For the comparison of therapeutic efficacy, binary data and continuous data were expressed as odds ratio or weighted mean difference, with a 95% CI. Heterogeneity was defined as the variability of research results. The significance level was set at *P* = 0.1. Where there was heterogeneity, a random-effects model was used; otherwise, a fixed-effects model was used.

Additionally, we used inconsistency factors to test the consistency of the closed loop and used the node-splitting method to evaluate the local inconsistency. In the “Results” section, the ranking probability of each intervention was expressed through a cumulative probability ranking graph, where the surface under the cumulative ranking curve (SUCRA) value was an index to summarize the cumulative ranking probability corresponding to the area under the curve of the probability graph, which is between 0 and 1. Higher values indicate greater therapeutic efficacy. All intervention measures were ranked based on the SUCRA value or the area under the curve, and the intervention measures were ranked. The 95% CI estimates and hypothesis test results of each variable are listed in the forest plots. RevMan 5.3 software was used to evaluate the publication bias of the included studies. In STATA version 16.0 Microsoft Windows software, we conducted a comprehensive network meta-analysis using the statistical software package Network and statistical package mvmeta.

## Results

### Search results

**Fig. 1:**
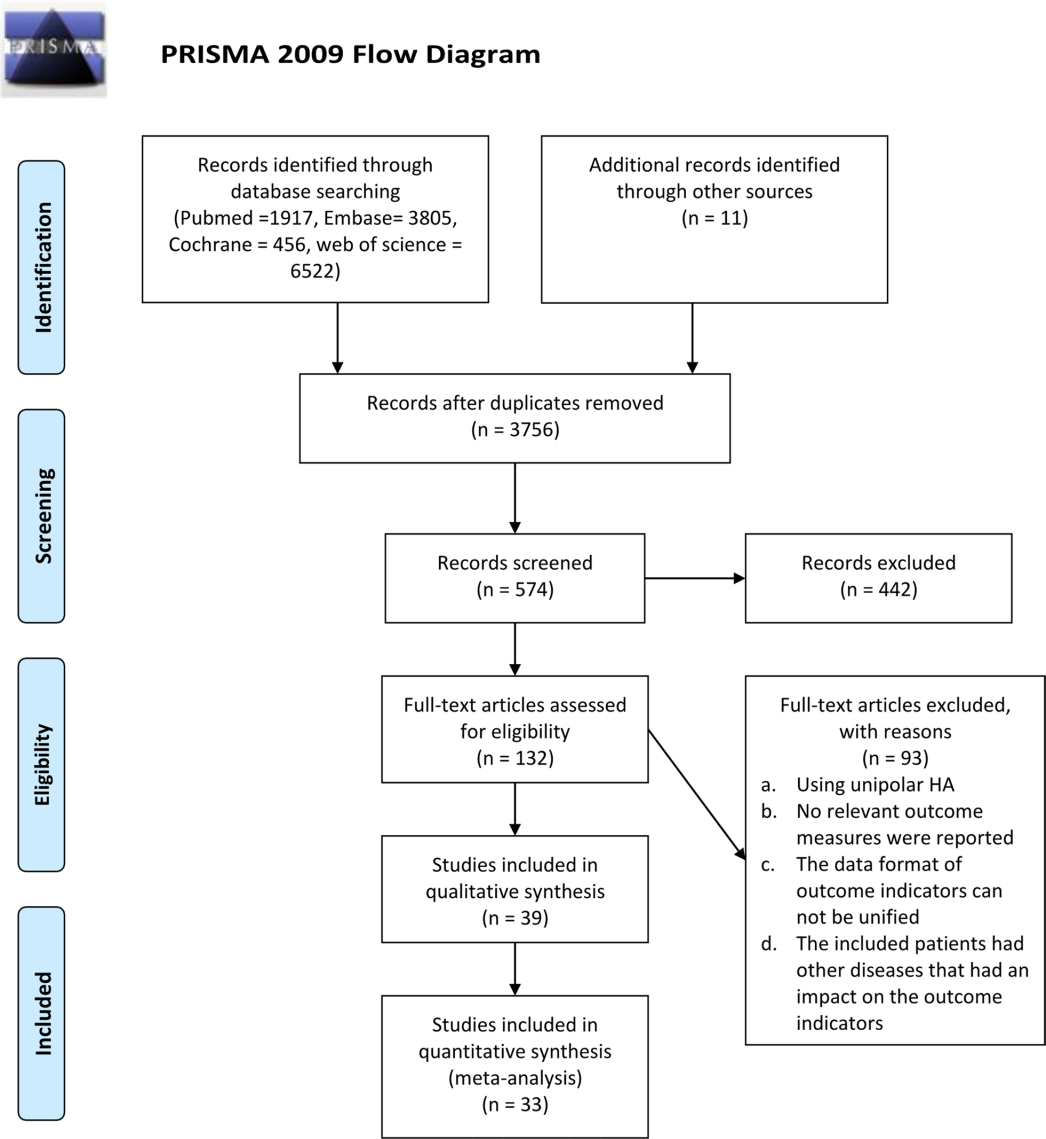
Flowchart of study selection

**Fig. 2:**
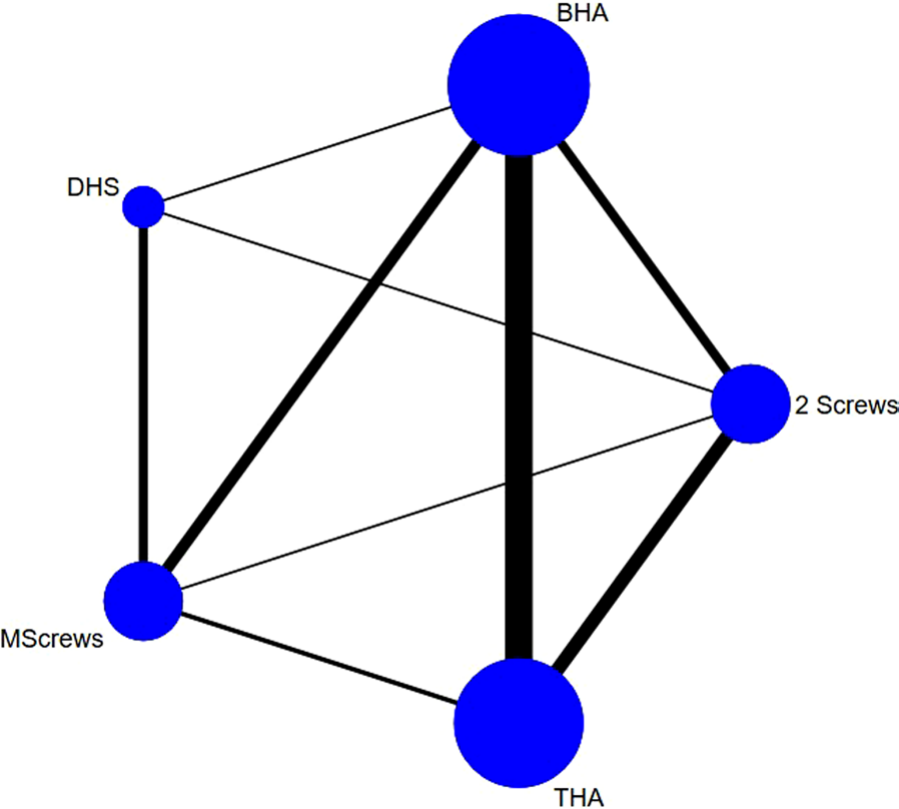
Direct comparisons in the network model. The thickness of the lines represents the number of studies; The size of the spots represent the number of patients (BHA = bipolar hemiarthroplasty; DHS = dynamic hip screw; THA = total hip arthroplasty)

A flowchart of the study selection in the present meta-analysis is shown in Fig. 1. The reasons for excluding trials or publications were documented. A total of 12,766 potential related studies were identified and screened for retrieval (Fig. 1). Finally, among 31 studies (67 arms), 3861 treated patients with femoral neck fractures were included in this meta-analysis. The main characteristics of the included trials are listed in Table 1. Figure 2 shows the network model comparing THA, BHA, double-screw internal fixation, multiple-screw internal fixation, and DHS surgical methods for repairing femoral neck fractures. (The thickness of the lines represents the number of studies; the blue spots represent the number of patients).

**Table 1:** Characteristics of selected trials

Study	Comparison			No. of patients	Age (years)	Female	Intervention or approach	Follow-up (months)	Lost to follow-up
Johansson [18]	2 Screws	THA		50/50	84/84	34/40	Two parallel and percutaneously inserted screws/Dorsolateral approach	180	NR
Iftikhar [19]	2 Screws	THA		50/50	65.16/65.04	26/34	Two cannulated screws/Posterolateral approach	18	NR
Chammout [20]	2 Screws	THA		57/43	79/78	41/38	Two cannulated screws/Posterolateral approach	204	23
Tidermark [21]	2 Screws	THA		53/49	81.4/79.2	42/40	two cannulated screws/Modified Hardinge approach THR	48	5
Lindström [22]	2 Screws	THA		50/50	84.0/84.2	38/40	Two parallel and percutaneously inserted screws/Dorsolateral approach	12	34
Neaiider [23]	2 Screws	THA		11/9	86/77	10/5	Two parallel Olmed screws/Posterolateral approach	18	NR
Cao [24]	MScrews	THA		128/157	76.8/75.9	69/84	Three hollow compression screws/Posterior approach	60	9
Kang [25]	MScrews	BHA		60/179	74.3/75.3	21/50	Three to four 6.5 mm cannulated screws/Posterolateral approach	36.8	NR
Kang [25]	MScrews	BHA		81/62	73.1/77.2	27/18	Three to four 6.5 mm cannulated screws/Posterolateral approach	36.8	NR
Frihagen [26]	2 Screws	BHA		112/110	83.2/82.5	84/78	Two parallel cannulated screws/Lateral approach	24	17
Dolatowski [27]	2 Screws	BHA		111/108	83.2/83.1	84/73	Two partially threaded, cancellous, cannulated screws of 8.0-mm diameter/Direct lateral approach or posterior approach	24	NR
Soreide [28]	2 Screws	BHA		51/53	77.9/78.3	38/43	Two von Bahr screws/Posterolateral modified Osborne approach	12	NR
Roden [29]	2 Screws	BHA		53/47	81/81	34/37	Bahr screws/Lateral approach	60	NR
Lu [30]	MScrews	BHA		41/37	85.85/86.24	29/29	Three 6.5 mm cannulated screws/Modified Hardinge approach	38.68	3
Vugt 1993[31]	DHS	BHA		21/22	75.3/76.0	11/14	DHS/Anterolateral approach	36	7
Keating [32]	MScrews	BHA	THA	69/69/69	74.3/75.0/75.2	51/54/52	Cannulated hip screws or a sliding hip screw/Lateral or posterior approach/Lateral or posterior approach	24	NR
Watson [33]	MScrews	DHS		23/26	76.7/77.9	24/25	Three partially threaded cannulated 6.5-mm titanium cancellous screws/Two-hole DHS	24	34
Lee [34]	MScrews	DHS		27/36	72.8/74.6	15/13	Three 6.5-mm (AO) cannulated screws/DHS	12	12
Jettoo [35]	MScrews	DHS		34 870/18 014	78/80	25,634/13343	Multiple screws/DHS	48	NR
Madsen [38]	MScrews	DHS		51/52	74/75	41/37	Multiple cannulated screws/DHS	24	0
Lagerby [39]	2 Screws	MScrews		138/130	81/80	93/86	Two Uppsala screws/Three Richards screws	12	0
Boukebous [40]	THA	BHA		98/101	78.8/83.3	70/73	Posterolateral approach	24.2	NR
Mariconda [41]	THA	BHA		60/60	75.8/78.8	49/48	Posterolateral or direct lateral approach/Direct lateral approach	12	0
Lin [42]	THA	BHA		115/96	64.1/67.9	58/52	NA	89.4	NR
Baker [43]	THA	BHA		40/41	74.2/75.83	32/32	Transgluteal lateral approach	36	NR
Kim [44]	THA	BHA		84/84	73.1/72.9	58/57	Posterolateral approach	21.9	0
Shukla [45]	THA	BHA		22/25	65.36/68.3	14/16	Standard Moore's (southern) posterior approach	24	NR
Cho [46]	THA	BHA		80/89	75.5/77.6	66/73	Modified Hardinge approach	36	NR
Sonaje [47]	THA	BHA		21/21	66.4/65.3	13/14	Lateral decubitus position approach	24	2
Blomfeldt [48]	THA	BHA		60/60	80.7/80.5	54/47	Modified Hardinge approach/Modified Hardinge approach	12	0
Cadossi [49]	THA	BHA		47/49	84.2/82.3	34/28	Straight lateral approach	36	64
Cans [50]	THA	BHA		16/22	82/77	NA	posterolateral approach/Lateral approach	25.2	8

### Primary results of the network meta-analysis

#### HHS score within six months after surgery

**Fig. 3:**
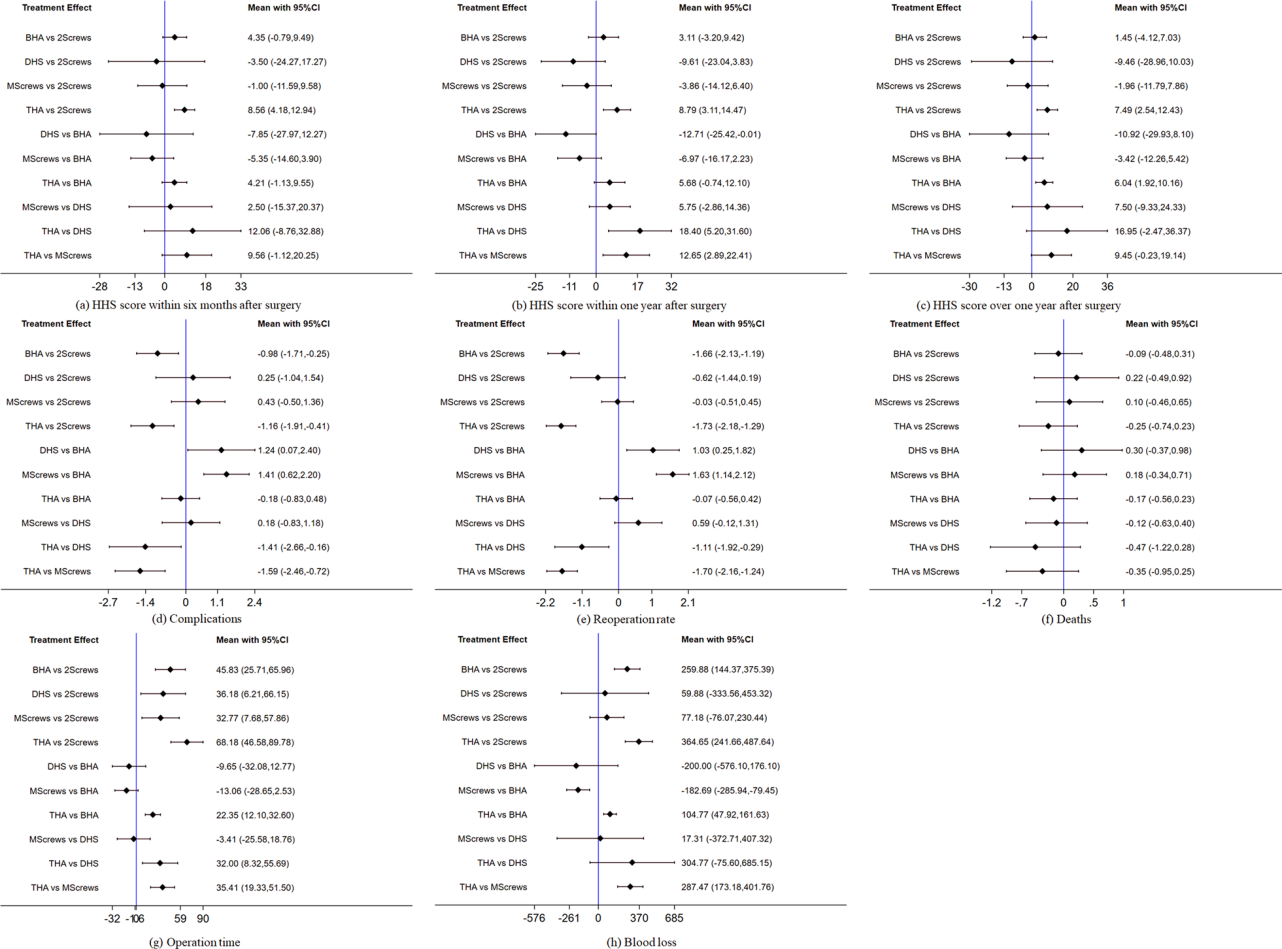
Final results of the network meta-analysis

Estimates of the therapeutic efficacy by HHS score are shown in Fig. 3a. In general, the HHS value of THA within six months was higher than that of other groups, but most comparison results did not reach statistical significance. Based on SUCRA method, the six-month HHS values for all surgical interventions, ranked from high to low, were as follows: THA, BHA, double-screw fixation, multiple-screw fixation, and dynamic hip screw (Table 2).

**Table 2:** Treatment measures ranked by SUCRA method

Treatment	HHS (within half a year)	HHS (1 year)	HHS (above 1 year)	Complications	Reoperation	Operative time	Blood loss
2Screws	31.2	46.3	45.0	36.5	12.9	99.7	86.6
BHA	66.7	69.7	58.5	82.0	84.5	31.2	28.9
DHS	28.2	5.0	13.3	25.4	47.0	54.4	67.1
MScrews	29.6	30.2	35.0	14.0	15.4	64.6	65.9
THA	94.3	95.4	98.2	92.1	90.3	0.1	1.6

**Fig. 4:**
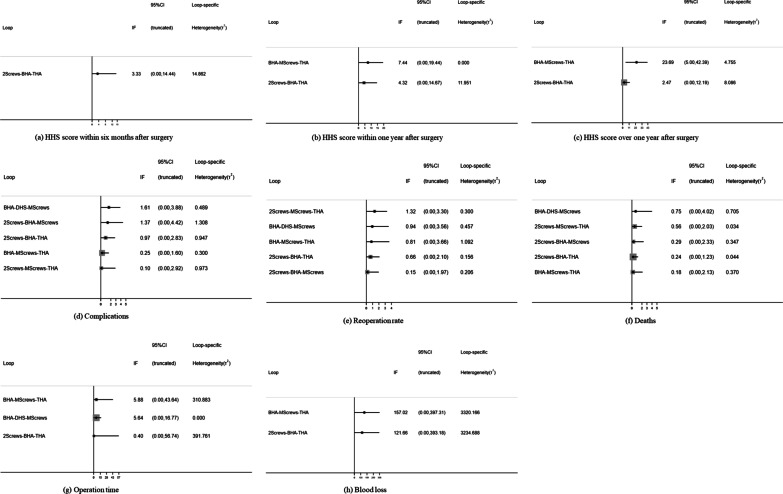
Inconsistency in the closed loops

The Z test was performed to test the inconsistency. No loop indicated the possibility of inconsistency (Fig. 4a).

#### HHS score within 1 year after surgery

Figure 3b demonstrates the estimated impact of different interventions on HHS within one year. The HHS value of THA within one year after surgery was higher than that of other groups, but the results of most comparisons were not significantly different, and no loop indicated the possibility of inconsistency (Fig. 4b).

The one-year HHS values for all the surgical interventions ranked from high to low were as follows: THA, BHA, double-screw fixation, multiple-screw fixation, and dynamic hip screw (Table 2).

#### HHS score over 1 year after surgery

As demonstrated in Fig. 3c, the HHS value of THA over 1 year after surgery was higher than that of other groups. The > 1-year HHS values for all the surgical interventions ranked from high to low were as follows: THA, BHA, double-screw fixation, multiple-screw fixation, and DHS (Table 2). There were potential inconsistencies in the loop of BHA, multiple-screw fixation, and THA (P = 0.013) resulting from a large difference in the follow-up times described in the included studies (Fig. 4c).

### Secondary results of the network meta-analysis

#### Main results of complications

Figure 3d indicates the estimated impact of different interventions on complications. There were some significant differences in complications associated with all the five surgical interventions for femoral neck fractures. Based on these results, the complication rates ranked from low to high were as follows: THA, BHA, double-screw fixation, DHS, and multiple-screw fixation (Table 2). The inconsistency in the closed loop was evaluated with the inconsistency factors (Fig. 4d), and no loop showed inconsistency. In particular, considering all cases in the included literature, the incidence of complications after three kinds of internal fixation was about 25.4%.

#### Main results of reoperation rate

There were some significant differences in the reoperation rate after five surgical procedures for femoral neck fractures (Fig. 3e). The rate of reoperation ranked from low to high was as follows: THA, BHA, DHS, multiple-screw fixation, and double-screw fixation (Table 2). The inconsistency factor was used to evaluate the inconsistency in the closed loop, and no loop indicated the possibility of inconsistency (Fig. 4e).

#### Main results of deaths

Overall, there was no significant difference between groups (Fig. 3f), so the mortality of different interventions could not be ranked. The inconsistency factor was used to evaluate the inconsistency in the closed loop, and there was no loop, indicating the possibility of inconsistency (Fig. 4f).

#### Main results of operation time

Figure 3g demonstrates the estimated impact of different interventions on the operation time. The operation time from short to long, as ranked by the SUCRA method, was as follows: double-screw fixation, multiple-screw fixation, DHS, BHA, and THA (Table 2). The inconsistency factor was used to evaluate the inconsistency in the closed loop, and no loop indicated the possibility of inconsistency (Fig. 4g).

#### Main results of intraoperative blood loss

Figure 3h shows the estimated impact of different interventions on intraoperative blood loss. The intraoperative blood loss ranked via the SUCRA method from low to high was as follows: double-screw fixation, DHS, multiple-screw fixation, BHA, and THA (Table 2). The inconsistency in the closed loop was evaluated using the inconsistency factors (Fig. 4h).

## Discussion

The choice of treatment methods for femoral neck fractures remains controversial. Here, we conducted a network meta-analysis of current relevant literature and considered as many outcome indicators as possible to discuss and sort the existing treatments for femoral neck fractures from all aspects, giving comprehensive recommendations to provide a basis for clinical decision-making.

For relatively healthy elderly patients with femoral neck fractures, the postoperative hip joint function is related to the quality of daily life after surgery, which is an important consideration in choosing proper surgical procedures. HHS is a commonly used index to evaluate the postoperative function of patients with femoral neck fractures. Postoperative HHS has been statistically significant in many meta-analyses [3, 4, 11]. In a study by Gao et al. [51], the HHS of patients undergoing joint replacement was generally higher than that of patients treated with internal fixations, which is consistent with the meta-analysis of Burgers et al. [52]. However, their studies only made a short follow-up comparison, which was not comprehensive. However, there was no significant difference between HHS scores after joint replacement and internal fixation under long-term follow-up [52].In this study, the three intervals for the follow-up examinations were short-term (within six months), mid-term (within 1 year), and long-term (more than 1 year). There was no significant difference in HHS between THA and other surgical procedures in the short-term follow-up examination, except for double-screw fixation. In the mid-term follow-up examination, the HHS score of BHA group was not significantly better than that of other internal fixation methods. In a comparison of internal fixation methods, Watson showed that multiple-screw fixation was significantly better upon a 1-year follow-up examination because the screw fixation was less invasive, the size of three small screw heads around the greater trochanter was smaller, and a larger dynamic hip system could cause fascia lata stimulation. In this paper, the results showed that there was no significant difference between double screw, multi-screw and DHS in HHS.A prospective study by Blomfeldt et al. [53] comparing HA and THA indicated a better functional outcome in THA during a 1-year follow-up examination. In the present study, we found no significant differences in THA and BHA at the short- and mid-term follow-up examinations, but THA was significantly better than BHA in the long-term follow-up examination.

Common complications of joint replacement surgery include periprosthetic fractures, aseptic loosening, and hip dislocation. Nonunion and ischemic osteonecrosis are two common complications of internal fixations. A meta-analysis [54] showed no statistical difference in deep infection and subsequent ipsilateral fractures between joint replacement and internal fixation at mid- and long-term follow-up examinations. In general, the incidence of postoperative complications of internal fixation is higher than that of arthroplasty, which is about 25.4%. Parker and Blundell [55] conducted a meta-analysis of randomized trials comparing different internal fixation methods for femoral neck fractures and did not find significant differences between different implants. Additionally, arthroplasty is usually associated with the risk of long-term complications, such as periprosthetic fractures and aseptic loosening [22]. Boukebous[40] demonstrated that THA, in contrast to BHA, is more suitable for weaker patients suffering from femoral neck fractures because of a lower incidence of postoperative complications, and this is consistent with the conclusion of our study. Jettoo et al. [35], in their retrospective study, reported that the avascular necrosis rate after sliding screw plate fixation was 3.2 times higher than that after multiple cancellous bone screw fixation. However, our study indicated that the overall postoperative complication rate of DHS was less than that of two-screw fixation, and there was no significant difference among other internal fixations.

Chammout et al. [58–60] supported the conclusion that internal fixations cause a greater reoperation rate. In the meta-analysis by Jin Jiang et al. [52], the percentages of patients undergoing reoperation during the mid- and long-term follow-up examinations were 6.5% (62/865) and 14.3% (69/483) in the joint replacement group, and 39.8% (336/844) and 43.8% (234/534) in the internal fixation group, respectively. Similar results were observed in our meta-analysis. Wang et al. [61] showed that, compared with THA, HA resulted in more reoperations, especially during long-term follow-up, attributed to acetabular erosion that generally occurred in the patient population in the fourth year after surgery. The present study indicated no significant difference in the rate of reoperation between BHA and THA, and BHA was significantly better than other surgical procedures. This indicates that BHA has similar potential to THA in reducing the risk of reoperation. Parker [55] conducted a meta-analysis that compared various internal fixation methods in 1998. They found that, compared with the reoperation rate, the complication rate is more suitable as an outcome indicator for comparisons between internal fixation methods. These results are consistent with those of our study, wherein no significant difference was found in the rate of reoperation between double screws, multiple screws, and DHS.

Blomfeldt et al. [62, 63] found that during THA, intraoperative blood loss and operation time were significantly increased compared with other surgical procedures. The present meta-analysis also indicated that the three types of internal fixations took less operation time and resulted in less blood loss than BHA and THA. However, Liodakis et al. [64] believed that more blood loss (100–150 mL) during THA did not increase the rate of general complications or mortality. Studies [65] have also shown that patients are more concerned about the risk of postoperative complications than the risk of intraoperative bleeding.

Bhandari et al. [66] demonstrated that, compared with artificial joint replacement, internal fixation had a significantly higher risk of death during the short-term follow-up. In our network meta-analysis, we found no difference in the long-term mortality of THA, HA, double-screw fixation, multiple-screw fixation, and DHS.

This study has some potential limitations. We did not discuss confounding factors involving surgical approaches and implant materials that may influence different results. As previously reported, surgical approaches for hip arthroplasty usually include direct anterior (Smith-Peterson), anterolateral (Watson-Jones), lateral (Hardinge), posterior (Moore), or posterolateral approaches [38, 61–64]. For internal fixation, the choice of internal fixation devices, the initial displacement, the degree of reduction, and the position of the internal fixation are of great significance to the healing of fractures.

## Conclusion

Generally, THA has a good performance for postoperative hip function, complication rate, and revision rate, but it has a longer operation time and results in a greater loss of blood during surgery. Therefore, patients should choose an appropriate surgical procedure based on their conditions to increase the chance of longer life and a higher quality of life.

## Supplementary Information

**Additional file 1.** PRISMA checklist.

## Data Availability

All the data and materials were included within the manuscript.

## Abbreviations

HA: Hemiarthroplasty
THA: Total hip arthroplasty
DHS: Dynamic hip screws
BHA: Bipolar hemiarthroplasty
HHS: Harris hip score
SD: Standard deviation
CI: Confidence interval
OR: Odds ratio
MD: Mean difference
SUCRA: Surface under the cumulative ranking curve
RCTs: Randomized controlled trials
