# Supplementary material for: Curative efficacy of surgical procedures for older patients with femoral neck fracture: a network meta-analysis and systematic review
Source: J Orthop Surg Res. 2022 Mar 2;17:127. doi: 10.1186/s13018-022-02914-y (PMC8889721; doi:10.1186/s13018-022-02914-y)
Supplement: Supplementary file 1 — Additional file 1. PRISMA checklist. [file 13018_2022_2914_MOESM1_ESM.doc]

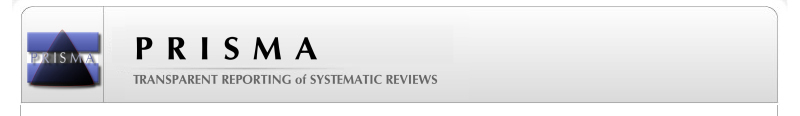
**PRISMA 2009 Flow Diagram**

**Screening**

**Included**

**Eligibility**

**Identification**

Records identified through database searching
(Pubmed =1917, Embase= 3805, Cochrane = 456, web of science = 6522)

Additional records identified through other sources
(n = 11)

Records after duplicates removed
(n = 3756)

Records screened
(n = 574)

Records excluded
(n = 442)

Full-text articles assessed for eligibility
(n = 132)

Full-text articles excluded, with reasons
(n = 93)

1. Using unipolar HA
2. No relevant outcome measures were reported
3. The data format of outcome indicators can not be unified
4. The included patients had other diseases that had an impact on the outcome indicators

Studies included in qualitative synthesis
(n = 39)

Studies included in quantitative synthesis (meta-analysis)
(n = 33)
